# Considerations for mitigating COVID-19 related risks in schools

**DOI:** 10.1016/j.lana.2021.100077

**Published:** 2021-09-10

**Authors:** Amy Gimma, Sham Lal

**Affiliations:** 1 London School of Hygiene & Tropical Medicine, Greater London UNITED KINGDOM

**Keywords:** Risk mitigation, COVID-19, School policy, Covid-19, Race, Social determinants

## Abstract

As the new school year begins in the United States, school districts will be tasked with providing in-person teaching while keeping children and school staff safe, an increasingly difficult goal in the presence of the COVID-19 delta variant. We aim to provide updated interpretations of past and newly published studies to assist in assessing risk in schools, and to add additional perspectives on addressing the social determinants of learning and on the role of race and other social factors. We advocate for the continued implementation of risk mitigation strategies in schools, including mandatory mask policies, improved ventilation, and convenient access to vaccinations for those eligible, as recommended by the CDC, and to use this opportunity to make long-term improvements to our schools as a matter of urgency.

## 1 Introduction

As schools open for the 2021 school year, communities face difficult decisions regarding the safety and well-being of children and school staff. During the previous school year, many parents and school staff demanded that districts only open schools when they had implementedrisk mitigation strategies in accordance with CDC guidelines. Similar demands have now followed through to the new school year and are once again being met with opposition from those advocating for full reopening without mitigation measures. In addition to academic offerings, school districts are eager to continue to safely provide valuable additional services to children and communities, including free meals, food pantry services, mental health counseling, and before and after school programs. Levison and colleagues [1] highlight the harms children suffer due to school closures and how barriers to educational achievement and psychosocial and emotional development are not experienced equally amongst all children and their families. Importantly, their commentary captures the harms children may face because of prolonged school closures by socio-demographic characteristics and constructs, such as race, social class, and urbanity. While the commentary contributes to the debate on COVID-19 policies in schools, key areas warrant further consideration.

## 2 Legitimate concerns among the education community

The success in reducing transmission using mitigation measures in schools has likely resulted in an underestimation of the potential contribution of the school to community infections. [2] There is evidence suggesting that these measures contributed to low estimated prevalence within schools and that symptom-based testing policies may have missed significant numbers of infections.[2] Global increases in cases have been reported in young adults and children, especially where the delta variant is present. [3] This variant is estimated to be more than twice as transmissible and may cause more severe infection, even, though rare, among otherwise healthy young adults and children. [3,4] While the vaccines are effective in preventing severe disease in most people, vaccinated individuals may still become infected and transmit to others. [5] The vaccinated are at a growing risk of becoming severely ill due to breakthrough infections [3,6] and may be at risk of waning immunity. [7] Vaccine hesitancy and misinformation is another crucial factor in transmission, and will affect the safety of schools for the foreseeable future.

The at-risk population of the school community includes both children and the education workforce. In addition to the approximately 5.5 million teachers and other instructors, about 3 million additional staff members would be at increased risk in the absence of mitigation measures. [8] The burden of risk is also carried by families and other close contacts of students and school staff, some of whom may be unvaccinated or vulnerable for other reasons. The risk of spreading COVID-19 within the educational community, their families, and the wider community is multi-faceted. We should not underestimate that risk in weighing the risks and benefits of children returning to school for in-person learning.

## 3 Addressing the social determinants of learning and health

The Levinson article [1] discusses the consequences of school closures by describing the harms experienced by racial groups. However, in documenting these disparities, it uses the terms families, communities, and people, “of color.” While it is important to consider the unique experiences of racial groups, collective groupings such as “people of color” suggest a binary separation between White people and non-White people. This grouping crudely aggregates those who identify as African American, Asian, Black, Native American, and Latino or Hispanic as the "other" people in American society. This substantially limits understanding of the harms experienced by each group and how these harms intersect with other social determinants of health and education. Social status, poverty, gender, religion, geography, the built environment, and household occupations are factors associated with race in the US that might also differentially drive disparities. Accounting for these factors frames the issue not in terms of the characteristics or actions of individuals, but to the conditions which inform their decisions, including a return to school.

We urge academics and policymakers to fully explore how the interconnectedness of race and other social determinants disproportionately impacts educational opportunities and outcomes, and frame these impacts within an intersectional theory that allows the public health and educational community to understand disparities between racial groups in relation to other social factors. [10] This could avoid the further marginalization of vulnerable groups and improve the uptake of public policy interventions during the pandemic. A richer understanding of these factors can provide critical information regarding the impacts of mitigation measures on different communities and their interconnectedness with other social circumstances. For example, disparities in healthcare have increasingly been described in terms of exposure to racism rather than as a function of race, which can be expanded on as a social determinant of learning. [9]

## 4 Reframing district and union negotiations

Teachers unions and district administrations in many areas have successfully negotiated (often in places where relationships between these stakeholders are fraught with tension) to partially or fully reopen by following CDC advice on school safety measures and building consensus on complex decisions, such as providing early access to vaccinations for teachers and school staff. [11,12] Some districts made accommodations for staff who feared transmitting the virus to vulnerable family members, negotiated more testing and absence coverage for illness, and amended teacher contracts through collective bargaining agreements.

Union activity is often characterized as antagonistic to the goals of returning children to schools. However, this past year shows that moving toward a shared goal of reopening schools can present a rare opportunity for collaboration and healthy debate, creating safer environments for all school community members.

## 5 An opportunity for long-term improvements and a path forward

Teachers’ unions and parents may, justifiably, be using this rare political and practical opportunity for explicitly and deliberately not returning to the status quo. Students across the country have endured schools with inadequate ventilation, poor sanitation, and crowded classrooms for generations. These issues are compounded by the intersectional issues of race and the social determinants of health and learning.

Throughout the first year of the pandemic, healthcare organizations received rapid funding for facility upgrades, medical workforce reinforcement, and updated infection control protocols. We need to extend the same level of funding and innovative processes to ensure the return to in-person education provides a safe learning, teaching, and working environment for all. We recommend prioritizing mandatory mask-wearing, robust vaccination campaigns, improved ventilation in classrooms, addressing wider safety issues (such as facility improvements and adequate staffing), regular and frequent testing for teachers and students, distancing for parents in or around the school, and a focus on hand hygiene.

Amid a public health crisis, communities are presented with a valuable opportunity to make lasting improvements to our schools and address long-standing inequities. This window of opportunity is not coincidental, with public health and education inseparably intertwined. Access to high-quality healthcare is a social determinant of learning, and access to education is a social determinant of health.

## Contributors

Conceptualization - AG and SL

Writing – original draft - AG

Writing – review & editing - AG and SL

## Declaration of interests

The authors declared no conflicts of interest.
